# Optimization and a Kinetic Study on the Acidic Hydrolysis of Dialkyl α-Hydroxybenzylphosphonates

**DOI:** 10.3390/molecules25173793

**Published:** 2020-08-20

**Authors:** Nikoletta Harsági, Zita Rádai, Áron Szigetvári, János Kóti, György Keglevich

**Affiliations:** 1 Department of Organic Chemistry and Technology, Budapest University of Technology and Economics, 1521 Budapest, Hungary; harsagi.nikoletta@mail.bme.hu (N.H.); radai.zita@mail.bme.hu (Z.R.); 2 Gedeon Richter Plc., 1475 Budapest, Hungary; szigetvaria@richter.hu (Á.S.); j.koti@richter.hu (J.K.)

**Keywords:** dialkyl α-hydroxyphosphonates, hydrolysis, phosphonic ester–acid intermediate, phosphonic acid, rate constants, mechanism

## Abstract

The two-step acidic hydrolysis of α-hydroxybenzylphosphonates and a few related derivatives was monitored in order to determine the kinetics and to map the reactivity of the differently substituted phosphonates in hydrolysis. Electron-withdrawing substituents increased the rate, while electron-releasing ones slowed down the reaction. Both hydrolysis steps were characterized by pseudo-first-order rate constants. The fission of the second P-O-C bond was found to be the rate-determining step.

## 1. Introduction

The hydrolysis of P-esters (e.g., phosphinates and phosphonates) resulting in the formation of the corresponding acids (phosphinic acids and phosphonic acids, respectively) is an important chemical transformation, and hence it is applied widely in syntheses. Most often, the hydrolyses were performed under acidic conditions [1,2,3,4], but the application of NaOH or KOH is also common [5,6,7,8]. An additional possibility is the fission of the P-O-C unit by the effect of Me_3_SiBr [9,10,11]. Usually, the acid- or base-catalyzed hydrolyses were carried out routinely, under “excessive” (unoptimized) conditions applying the acid or base catalysts in a larger quantity than required, and allowing longer reaction times. We undertook to explore the optimum conditions for the HCl-catalyzed hydrolysis of phosphinic and phosphonic esters. In the first round, the acid-catalyzed hydrolysis of cyclic phosphinates, such as 1-alkoxy-3-phospholene oxides, 1-alkoxyphospholane oxides, and an 1-alkoxy-1,2,3,4,5,6-hexahydrophosphinine oxide was investigated, optimized, and characterized by rate constants [12]. Then, the hydrolysis of a series of dialkyl arylphosphonates was studied. In this case, two-step conversions were monitored and quantified by k values [13]. α-Hydroxybenzylyphosphonates, obtained in the Pudovik reaction of substituted benzaldehydes and dialkyl phosphites, form a representative class of phosphonic acid derivatives [14]. α-Hydroxyphosphonates are versatile intermediates that may be transformed to α-aminophosphonates [15], can be phosphorylated [16], and may be rearranged to the corresponding phosphates [17]. The catalytic hydrogenation of α-dibenzyl hydroxyphosphonates afforded the respective α-hydroxyphosphonic acids [18]. Moreover, they may be of cytotoxic activity [18]. In this article, we describe our results on the HCl–promoted hydrolysis of α-hydroxyphosphonates and a few related analogues.

## 2. Results and Discussion

**Scheme 1:**
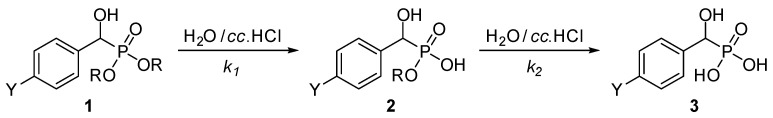
Two-step acidic hydrolysis of substituted α-hydroxybenzylphosphonates. For the Y substituents see Table 1.

**Figure 1:**
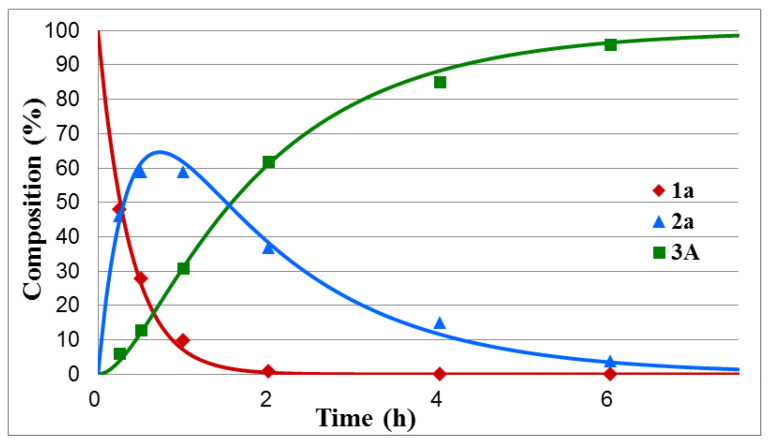
Concentration profile for the components during the hydrolysis of dimethyl α-hydroxybenzylphosphonate (**1a**) under optimum conditions. The R^2^ measure of goodness of fit is 0.994.

**Figure 2:**
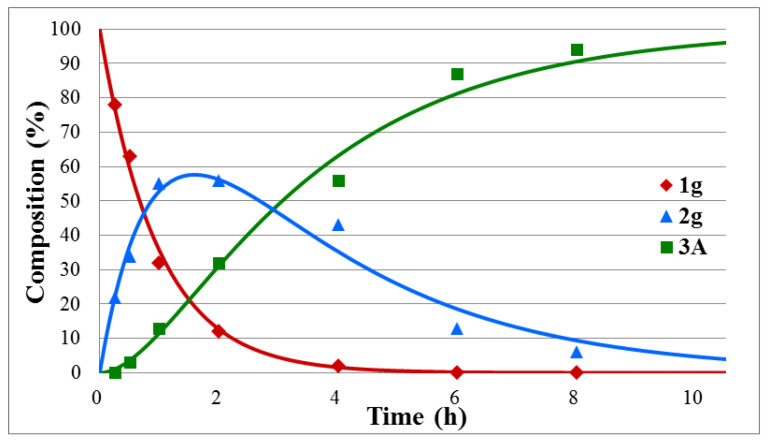
Concentration profile for the components during the hydrolysis of diethyl α-hydroxybenzylphosphonate (**1g**) under optimum conditions. The R^2^ measure of goodness of fit is 0.986.

Substituted α-hydroxybenzylphosphonates (**1a**–**j**) prepared as described earlier [18] were subjected to acidic hydrolysis. The application of three equivalents (0.5 mL) of concentrated hydrochloric acid in 1 mL of water for ca. 2 mmol of the phosphonate (**1**) at reflux resulted in complete hydrolysis within 2.5–9.5 h depending on the substituents. The reactions followed a two-step protocol and took place via the corresponding ester–acid intermediate **2**, and were monitored by ^31^P NMR spectroscopy (Scheme 1). Experimental data together with the calculated pseudo-first-order k_1_ and k_2_ rate constants are listed in Table 1, while the concentration–time diagrams exhibiting the relative proportions of components **1**, **2**, and **3** are shown in Figure 1 and Figure 2 and Figures S1–S8 in the Supplementary Materials.

One can see that the hydrolysis of the unsubstituted dimethyl α-hydroxybenzylphosphonate was complete after t_r_ = 6.5 h, and the maximum proportion of intermediate **2a** could be observed at *t_max_* = 44 min. In this case, k_1_ and k_2_ were found to be 2.64 h^−1^ and 0.60 h^−1^, respectively (Table 1/Entry 1). Electron-withdrawing substituents, such as 4-NO_2_, 4-Cl and 4-F in the phenyl ring facilitated the hydrolyses that were complete after 2.5 h, 5.5 h, and 6 h, respectively. The maximum concentration of intermediates **2b**–**d** appeared in the range of 22–34 min. The k_1_ values fell in the range of 3.36–5.18 h^−1^, while the k_2_ constants were between 0.67 and 1.24 h^−1^ (Table 1/Entries 2–4). It seems that the 4-CF_3_Ph substituent acted overall as the 4-ClPh group, as marked by t_r_ = 5.5 h. In this case (Y = CF_3_), k_1_ was 2.03, while k_2_ was 0.61 (Table 1/Entry 5). In the above series, the hydrolysis of the 4-Me-substituted benzylphosphonate (**1f**) was the slowest, as a complete hydrolysis required 8 h, and the rate constants were found to be 1.64 (k_1_) and 0.31 (k_2_) (Table 1/Entry 6). It is noteworthy that the fission of the second P–OMe unit is the rate-determining step. As can be seen from Table 1, the k_1_ values for the above cases are, in almost all cases, more than four times larger as compared to the k_2_ values.

Regarding the series of substituted diethyl α-hydroxybenzylphosphonates (**1g**–**j**), the hydrolysis of the unsubstituted model (**1g**) was significantly slower than that of the dimethyl analogue (**1a**) (compare the reaction times of 9.5 h (Table 1/Entry 7) and 6.5 h (Table 1/Entry 1)). The corresponding k_1_ and k_2_ rate constants for the hydrolysis of diethyl ester **1g** were roughly the half the ones obtained for the methyl counterpart (**1a**) (compare rate constants 1.03/0.35 versus 2.64/0.60 (Table 1/Entry 7 versus Entry 1). Hydrolysis of the α-hydroxyphosphonates with electron-withdrawing 4-NO_2_, 4-Cl, and 4-F substituents in the phenyl ring (**1h-j**) required shorter reaction times of 5.5–9.0 h as compared with that (9.5 h) of the unsubstituted instance (**1g**) (Table 1/Entries 8–10). In these cases again, the k_2_ rate constants (0.61, 0.42, and 0.31, respectively) determined the overall reactivity.

**Scheme 2:**
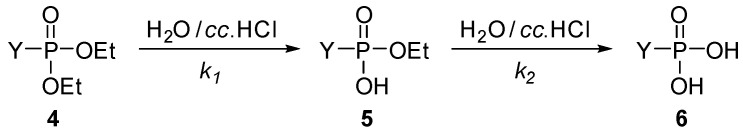
The two-step hydrolysis of other phosphonate derivatives. For the Y substituents see Table 2.

**Figure 3:**
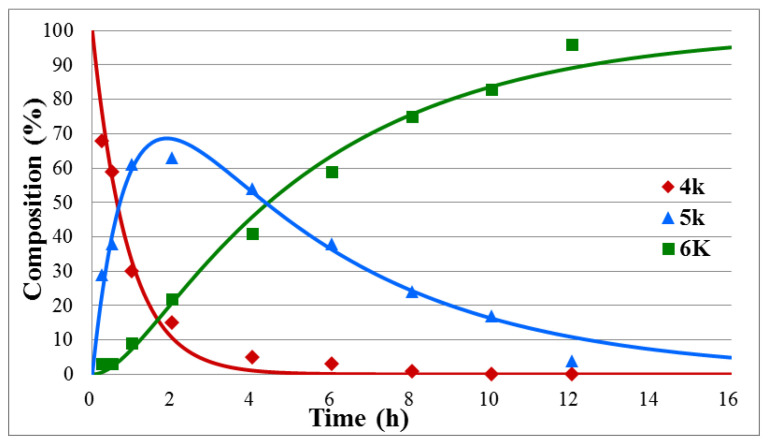
Concentration profile for the components during the hydrolysis of diethyl benzylphosphonate (**4k**) under optimum conditions. The R^2^ measure of goodness of fit is 0.983.

To study the effect of substituents on the rate of the hydrolysis further, three additional model compounds, diethyl benzylphosphonate (**4k**), diethyl α-phenylethylphosphonate (**4l**), and diethyl β-phenylethylphosphonate (**4m**) were also subjected to hydrolysis, under the conditions applied for the α-hydroxybenzylphosphonates (**1a**–**j**) above (Scheme 2, Table 2, Figure 3 and Figures S9 and S10 in Supplementary Materials). It was found that the hydrolysis of the benzylphosphonate (**4k**) took longer than that of the α-hydroxy derivative **1g** (15 h versus 9.5 h, Table 2/Entry 1 and Table 1/Entry 7). The k_1_ constant was somewhat larger for the hydrolysis of species **4k** than that for **1g**, but the decisive k_2_ value become lower, as demonstrated by 1.12 h^−1^ and 0.20 h^−1^ versus 1.03 h^−1^ and 0.35 h^−1^ data pairs, respectively. Placing a Me group instead of the OH function on the α C atom, i.e., starting from α-phenylethylphosphonate **4l**, the hydrolysis became even slower, and it was complete only after 25 h. The smallest k values (k_1_ = 0.51 h^−1^, k_2_ = 0.11 h^−1^) were obtained in this case (Table 2/Entry 2). It is obvious that the lack of the electron-withdrawing OH group in position α, or the appearance of an Me group instead of the HO function decreases the electrophilicity of the P atom of the P=O-function. The hydrolysis of β-phenylethylphosphonate (**4m**) with a reaction time of 20 h and k values of 0.70 h^−1^ and 0.15 h^−1^ (Table 2/Entry 3) occupied an intermediate position.

It is noted that the hydrolyses of the phosphonate function take place via the S_N_2 mechanism, i.e., by the nucleophilic attack of the water molecule on the P=O function. In the consecutive series, the fission of the second P-O-C bond is the rate-determining step.

The overall order of reactivity of the phosphonates (**1a**–**j** and **4k**–**m**) observed under acidic conditions was summarized in Table 3.

## 3. Materials and Methods

### 3.1. General Information

The ^31^P, ^13^C, and ^1^H NMR spectra were taken on a Bruker DRX-500 spectrometer operating at 202.4, 125.7, and 500 MHz, respectively. The couplings are given in Hz. LC-MS measurements were performed with an Agilent 1200 liquid chromatography system coupled with a 6130 quadrupole mass spectrometer equipped with an ESI ion source (Agilent Technologies, Palo Alto, CA, USA). High-resolution mass spectrometric measurements were performed using a Thermo Velos Pro Orbitrap Elite hybrid mass spectrometer in positive electrospray mode.

### 3.2. Use of the ^31^P NMR Spectra in Quantitative Analysis

The composition of the reaction mixture was determined by the integration of the areas under the corresponding peaks of the starting material, intermediate, and product in the ^31^P NMR spectra.

### 3.3. Curve Fitting on the Time–Relative Quantity Data Pairs

The acidic hydrolysis was modeled assuming pseudo-first-order kinetics. The concentration of water and hydrochloric acid was constant during the reaction, and their initial concentration is incorporated in the pseudo-first-order rate constants *k*_1_ and *k*_2_. The corresponding differential equations used in the model are the following:

(1) $$\frac{d\left[\mathrm{diester}\right]}{dt}=-k_{1}\left[\mathrm{diester}\right]$$

(2) $$\frac{d\left[\mathrm{ester}\;-\;\mathrm{acid}\right]}{dt}=k_{1}\left[\mathrm{diester}\right]-k_{2}\left[\mathrm{ester}\;-\;\mathrm{acid}\right]$$

(3) $$\frac{d\left[\mathrm{acid}\right]}{dt}=k_{2}\left[\mathrm{ester}\;-\;\mathrm{acid}\right]$$

where [diester], [ester-acid], and [acid] are the time-dependent molarities of the dialkyl α-hydroxyphosphonate, the phosphonic ester–acid intermediate, and the phosphonic acid, respectively, and *k*_1_ and *k*_2_ are the pseudo-first-order rate constants of the first and the second step of the hydrolysis.

The solution of the differential equations is the following (*k*_1_ ≠ *k*_2_):

(4) $$\left[\mathrm{diester}\right]=c_{0}e^{-k_{1}t}$$

(5) $$\left[\mathrm{ester}\;-\;\mathrm{acid}\right]=c_{0}\left(\frac{k_{1}}{k_{2}-k_{1}}e^{-k_{1}t}-\frac{k_{1}}{k_{2}-k_{1}}e^{-k_{2}t}\right)$$

(6) $$\left[\mathrm{acid}\right]=c_{0}\;\left(1-\frac{k_{2}}{k_{2}-k_{1}}e^{-k_{1}t}+\frac{k_{1}}{k_{2}-k_{1}}e^{-k_{2}t}\right)=c_{0}-\left[\mathrm{diester}\right]-\left[\mathrm{ester}\;-\;\mathrm{acid}\right]$$

where *c*_0_ is the initial molarity of the dialkyl α-hydroxyphosphonate.

The relative quantity of the components is their molarity divided by the sum of the three molarities (which is *c*_0_). The calculated time–composition curves are described by the following equations, by leaving the *k*_1_ and *k*_2_ rate constants as parameters:

(7) $${\mathrm{diester}}_{calcd}=e^{-k_{1}t}\times 100\%$$

(8) $$\mathrm{ester}\;-{\;\mathrm{acid}}_{calcd}=\frac{k_{1}}{k_{2}-k_{1}}\left(e^{-k_{1}t}-e^{-k_{2}t}\right)\times 100\%$$

(9) $${\mathrm{acid}}_{calcd}=100\%-{\mathrm{diester}}_{calcd}-\;\mathrm{ester}\;-{\;\mathrm{acid}}_{calcd}.$$

During the calculation of the rate constants, we first gave arbitrary initial values to *k*_1_ and *k*_2_, then, we optimized their values such that the sum of the squares of the differences between the experimental and calculated compositions became minimal:

(10) $$\begin{array}{ll} SS_{res} & =\overset{n}{\underset{i=1}{\sum }}{\left({\mathrm{diester}}_{\exp,i}-{\mathrm{diester}}_{\mathrm{calcd},i}\right)}^{2}\\ & +\overset{n}{\underset{i=1}{\sum }}{\left(\mathrm{ester}\;-{\;\mathrm{acid}}_{\exp,i}-\mathrm{ester}\;-{\;\mathrm{acid}}_{\mathrm{calcd},i}\right)}^{2}\\ & +\overset{n}{\underset{i=1}{\sum }}{\left({\mathrm{acid}}_{\exp,i}-{\mathrm{acid}}_{\mathrm{calcd},i}\right)}^{2}\to \min \end{array}$$

where *n* is the number of experimental time–composition data points measured at reaction times *t*_1_, *t*_2_, …, *t_n_*.

The resulting *k*_1_ and *k*_2_ rate constants and the associated time–composition curves were considered as the best fits. The best fits were found iteratively, using the nonlinear generalized reduced gradient method [19] of Microsoft Excel Solver.

The R^2^ measure of goodness of fit is calculated as $R^{2}=1-\left(\frac{SS_{res}}{SS_{tot}}\right)$, where *SS_res_* is described above and

(11) $$\begin{array}{ll} SS_{tot} & =\sum_{j=1}^{n}{\left({\mathrm{diester}}_{\exp,j}-\frac{1}{n}\sum_{i=1}^{n}{\mathrm{diester}}_{\exp,i}\right)}^{2}\\ & +\sum_{i=1}^{n}{\left(\mathrm{ester}-{\mathrm{acid}}_{\exp,j}-\frac{1}{n}\sum_{i=1}^{n}\mathrm{ester}-{\mathrm{acid}}_{\exp,i}\right)}^{2}\\ & +\sum_{j=1}^{n}{\left({\mathrm{acid}}_{\exp,j}-\frac{1}{n}\sum_{i=1}^{n}{\mathrm{acid}}_{\exp,j}\right)}^{2} \end{array}$$

The reaction time (*t_max_*) corresponding to the maximal ratio of the phosphonic ester–acid intermediate in the reaction mixture was found as follows:

(12) $$0={\frac{d\left[\mathrm{ester}\;-{\;\mathrm{acid}}_{calcd}\right]}{dt}|}_{t_{max}}={\frac{d}{dt}\left(\frac{k_{1}}{k_{2}-k_{1}}\left(e^{-k_{1}t}-e^{-k_{2}t}\right)\times 100\%\right)|}_{t_{max}}$$

(13) $$0={\frac{d}{dt}\left(e^{-k_{1}t}-e^{-k_{2}t}\right)|}_{t_{max}}=-k_{1}e^{-k_{1}t_{max}}+k_{2}e^{-k_{2}t_{max}}$$

(14) $$k_{1}e^{-k_{1}t_{max}}=k_{2}e^{-k_{2}t_{max}}$$

(15) $$\frac{k_{1}}{k_{2}}=\frac{e^{-k_{2}t_{max}}}{e^{-k_{1}t_{max}}}\equiv e^{\left(k_{1}-k_{2}\right)t_{max}}$$

(16) $$t_{max}=\frac{\ln\left(\frac{k_{1}}{k_{2}}\right)}{k_{1}-k_{2}}.$$

### 3.4. General Procedure for the Hydrolysis of Phosphonates (***1a***–***j***, ***4k***–***m***)

A mixture of 3.8 mmol of phosphonate (**1a**: 0.82 g, **1b**: 0.99 g, **1c**: 0.95 g, **1d**: 0.89 g, **1e**: 1.1 g, **1f**: 0.87 g, **1g**: 0.93 g, **1h**: 1.1 g, **1i**: 1.1 g, **1j**: 1.0 g, **4k**: 0.87 g, **4l**: 0.92 g, **4m**: 0.92 g), 1.0 mL (6.0 mmol) of *cc.* hydrochloric acid, and 2.0 mL of water was stirred at reflux for 2.5–25 h. The concentration of an aliquot part of the reaction mixture, or the whole mixture, afforded an oil that was analyzed by ^31^P NMR spectroscopy and LC-MS. Identification of the starting materials (**1a**–**j**, **4k**–**m**), intermediates (**2a**–**j**, **5k**–**m**), and products (**3A**–**F**, **6K**–**M**) can be found in Table 4. The ^13^C and ^1^H NMR spectral data of the new intermediates (**2a**–**f**, **h**–**j**) were obtained from the spectra of the corresponding mixtures containing also the phosphonic acids (**3A**–**F**).

^13^C and ^1^H NMR characterization of the new ester acids:

*Methyl hydrogen 1-(4-nitrophenyl)-1-hydroxymethylphosphonate* (**2b**). ^13^C NMR (DMSO-*d*_6_) δ: 52.6 (d, ^2^*J* = 6.5, OCH_3_), 69.1 (d, ^1^*J* = 157.5, PCH), 122.7 (d, ^4^*J* = 2.4, C_3_), 128.1 (d, ^3^*J* = 5.0, C_2_), 146.5 (d, ^5^*J* = 3.3, C_4_), 147.6 (C_1_); ^1^H NMR (DMSO *d*_6_) δ: 3.58 (d, ^3^*J* = 10.4, 3H, OMe), 5.05 (d, ^2^*J* = 15.5, 1H, PCH), 7.64–7.73 (m, 2H, H_2_), 8.17–8.23 (m, 2H, H_3_).

*Methyl hydrogen 1-(4-chlorophenyl)-1-hydroxymethylphosphonate* (**2c**). ^13^C NMR (DMSO-*d*_6_) δ: 53.0 (d, ^2^*J* = 6.5, OCH_3_), 69.4 (d, ^1^*J* = 160.3, PCH), 128.1 (d, ^4^*J* = 2.2, C_3_), 129.5 (d, ^3^*J* = 5.3, C_2_), 132.1 (d, ^5^*J* = 3.6, C_4_), 139.0 (C_1_); ^1^H NMR (DMSO-*d*_6_) δ: 3.55 (d, ^3^*J* = 10.3, 3H, OMe), 4.85 (d, ^2^*J* = 13.8, 1H, PCH), 7.32–7.47 (m, 4H, Ar).

*Methyl hydrogen 1-(4-fluorophenyl)-1-hydroxymethylphosphonate* (**2d**). ^13^C NMR (DMSO-*d*_6_) δ: 53.0 (d, ^2^*J*_P,C_ = 6.5, OCH_3_), 69.4 (d, ^1^*J*_P,C_ = 161.3, PCH), 114.9 (dd, ^2^*J*_F,C_ = 21.2, ^4^*J*_P,C_ = 1.9, C_3_), 129.7 (dd, ^3^*J*_F,C_ = 8.0, ^3^*J*_P,C_ = 5.6, C_2_), 136.1 (d, ^4^*J*_F,C_ = 2.6), 161.9 (dd, ^1^*J*_F,C_ = 242.5, ^5^*J*_P,C_ = 3.0, C_4_); ^1^H NMR (DMSO-*d*_6_) δ: 3.54 (d, ^3^*J* = 10.2, 3H, OMe), 4.84 (d, ^2^*J* = 13.2, 1H, PCH), 7.09–7.19 (m, 2H, H_3_), 7.38–7.49 (m, 2H, H_2_).

*Methyl hydrogen 1-(4-trifluoromethylphenyl)-1-hydroxymethylphosphonate* (**2e**). ^13^C NMR (DMSO-*d*_6_) δ: 53.1 (d, ^2^*J*_P,C_ = 6.5, OCH_3_), 69.7 (d, ^1^*J*_P,C_ = 157.9, PCH), 124.7–125.0 (m, C_3_), 124.9 (q, ^1^*J*_F,C_ = 271.1, CF_3_), 127.3–128.7 (m, C_2_, C_4_), 145.0 (C_1_); ^1^H NMR (DMSO-*d*_6_) δ: 3.56 (d, ^3^*J* = 10.3, 3H, OMe), 4.96 (d, ^2^*J* = 14.6, 1H, PCH), 7.57–7.72 (m, 4H, Ar).

*Methyl hydrogen 1-(4-methylphenyl)-1-hydroxymethylphosphonate* (**2f**). ^13^C NMR (DMSO-*d*_6_) δ: 21.2 (Ar-CH_3_), 52.8 (d, ^2^*J*_P,C_ = 6.6, OCH_3_), 70.0 (d, ^1^*J*_P,C_ = 160.2, PCH), 127.7 (d, ^3^*J* = 5.6, C_2_), 128.7 (d, ^4^*J* = 2.1, C_3_), 136.5 (d, ^5^*J* = 3.1, C_4_), 136.9 (C_1_); ^1^H NMR (DMSO-*d*_6_) δ: 2.28 (s, 3H, Ar-CH_3_), 3.52 (d, ^3^*J* = 10.2, 3H, OMe), 4.76 (d, ^2^*J* = 13.0, 1H, PCH), 7.06–7.38 (m, 4H, Ar).

*Ethyl hydrogen 1-(4-nitrophenyl)-1-hydroxymethylphosphonate* (**2h**) ^13^C NMR (DMSO-*d*_6_) δ: 16.9 (d, ^3^*J* = 5.6, CH_3_), 62.0 (d, ^2^*J* = 6.5, OCH_2_), 69.1 (d, ^1^*J* = 157.5, PCH), 122.7 (d, ^4^*J* = 2.4, C_3_), 128.2 (d, ^3^*J* = 4.9, C_2_), 146.5 (d, ^5^*J* = 3.6, C_4_), 147.6 (C_1_); ^1^H NMR (DMSO-*d*_6_) δ: 1.17 (t, ^3^*J*_H,H_ = 7.0, 3H, CH_3_), 3.91 (dq, ^3^*J*_P,H_ = 8.1, ^3^*J*_H,H_ = 7.0, 2H, OCH_2_), 5.03 (d, ^2^*J* = 15.7, 1H, PCH), 7.65–7.72 (m, 2H, H_2_), 8.16–8.23 (m, 2H, H_3_).

*Ethyl hydrogen 1-(4-chlorophenyl)-1-hydroxymethylphosphonate* (**2i**). ^13^C NMR (DMSO-*d*_6_) δ: 16.6 (d, ^3^*J* = 5.5, CH_3_), 61.7 (d, ^2^*J* = 6.5, OCH_2_), 69.3 (d, ^1^*J* = 160.7, PCH), 127.7 (d, ^4^*J* = 1.9, C_3_), 129.2 (d, ^3^*J* = 5.3, C_2_), 131.8 (d, ^5^*J* = 3.7, C_4_), 138.6 (C_1_); ^1^H NMR (DMSO-*d*_6_) δ: 1.15 (t, ^3^*J*_H,H_ = 7.1, 3H, CH_3_), 3.91 (dq, ^3^*J*_P,H_ = 7.1, ^3^*J*_H,H_ = 7.1, 2H, OCH_2_), 4.84 (d, ^2^*J* = 13.8, 1H, PCH), 7.33–7.47 (m, 4H, Ar).

*Ethyl hydrogen 1-(4-fluorophenyl)-1-hydroxymethylphosphonate* (**2j**). ^13^C NMR (DMSO-*d*_6_) δ: 16.9 (d, ^3^*J* = 5.6, CH_3_), 61.9 (d, ^2^*J* = 6.5, OCH_2_), 69.5 (d, ^1^*J*_P,C_ = 161.7, PCH), 114.8 (dd, ^2^*J*_F,C_ = 21.1, ^4^*J*_P,C_ = 2.0, C_3_), 129.7 (dd, ^3^*J*_F,C_ = 8.2, ^3^*J*_P,C_ = 5.5, C_2_), 136.1 (d, ^4^*J*_F,C_ = 2.8), 161.9 (dd, ^1^*J*_F,C_ = 242.4, ^5^*J*_P,C_ = 3.2, C_4_); ^1^H NMR (DMSO-*d*_6_) δ: 1.15 (t, ^3^*J*_H,H_ = 7.0, 3H, CH_3_), 3.87–3.95 (m, 2H, OCH_2_), 4.82 (d, ^2^*J* = 13.3, 1H, PCH), 7.11–7.17 (m, 2H, H_3_), 7.42–7.47 (m, 2H, H_2_).

*1-(4-Trifluoromethylphenyl)-1-hydroxymethylphosphonic acid* (**3e**). ^13^C NMR (DMSO-*d*_6_) δ: 70.5 (d, ^1^*J*_P,C_ = 157.7, PCH), 124.7–125.0 (m, C_3_), 124.9 (q, ^1^*J*_F,C_ = 272.3, CF_3_), 127.2–128.6 (m, C_4_), 128.4 (d, ^3^*J*_P,C_ = 5.0, C_2_), 145.6 (C_1_); ^1^H NMR (DMSO-*d*_6_) δ: 4.80 (d, ^2^*J* = 15.0, 1H, PCH), 7.55–7.72 (m, 4H, Ar).

## 4. Conclusions

Kinetic study of the two-step acidic hydrolysis of a series of dialkyl α-hydroxybenzylphosphonates and a few related model compounds allowed the mapping of the reactivity of the different substrates. The two-step hydrolyses were characterized by k_1_ and k_2_ pseudo-first-order rate constants belonging to the formation of the corresponding monoester monoacids and the phosphonic acids, respectively. Electron-withdrawing substituents increased the rate, while electron-releasing ones slowed down the hydrolyses starting with the nucleophilic attack of the water molecule. It turned out that the fission of the second P-O-C unit is the rate-determining step. The intermediate ester–acid species were identified and characterized.

## Figures and Tables

**Table 1:** Experimental and kinetic data on the two-step hydrolysis of α-hydroxybenzylphosphonates **1a**–**j**.

Entry	Y	R	*t_max_* (min)	t_r_ (h)	k_1_ (h^−1^)	k_2_ (h^−1^)	R^2^
1	H (**a**)	Me	44	6.5	2.64	0.60	0.994
2	NO_2_ (**b**)	Me	22	2.5	5.18	1.24	0.989
3	Cl (**c**)	Me	34	5.5	3.36	0.79	0.987
4	F (**d**)	Me	32	6.0	3.93	0.67	0.965
5	CF_3_ (**e**)	Me	51	5.5	2.03	0.61	0.988
6	Me (**f**)	Me	76	8	1.64	0.31	0.962
7	H (**g**)	Et	90	9.5	1.03	0.35	0.986
8	NO_2_ (**h**)	Et	75	5.5	1.40	0.61	0.992
9	Cl (**i**)	Et	60	8.0	1.08	0.42	0.992
10	F (**j**)	Et	80	9.0	1.35	0.31	0.970

**Table 2:** Experimental and kinetic data on the two-step hydrolysis of phosphonates **4k**–**m**.

Entry	Y	*t_max_* (h)	t_r_ (h)	k_1_ (h^−1^)	k_2_ (h^−1^)	R^2^
1	PhCH_2_ (**k**)	2	15	1.12	0.20	0.983
2	PhCHMe (**l**)	4	25	0.51	0.11	0.940
3	Ph(CH_2_)_2_ (**m**)	2.75	20	0.70	0.15	0.949

**Table 3:** Reactivity order of the phosphonates **1a**–**j** and **4k**–**m** in acidic hydrolyses characterized by t_r_, as well as k_1_ and k_2_.

	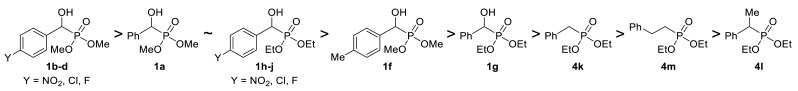							
t_r_ (h)	2.5–6.0	6.5	5.5–9.0	8	9.5	15	20	25
k_1_ (h^−1^)	3.36–5.18	2.64	1.08–1.40	1.64	1.03	1.12	0.70	0.51
k_2_ (h^−1^)	0.67–1.24	0.60	0.31–0.61	0.31	0.35	0.20	0.15	0.11

**Table 4:** Identification of the starting phosphonates (**1a**–**j**, **4k**–**m**), ester–acid intermediates (**2a**–**j**, **5k**–**m**), and phosphonic acids (**3A**–**F**, **6K**–**M**).

	δ ^31^P NMR		[M + H]
	Found (DMSO)	Literature	
**1a**	23.9	23.9 (CDCl_3_) [20]	216.9
**1b**	22.7	21.9 (CDCl_3_) [20]	262.2
**1c**	23.5	22.1 (CDCl_3_) [20]	273.0
**1d**	23.8	23.3 (CDCl_3_) [21]	235.0
**1e** [22] ^a^	23.1	–	285.1
**1f**	24.1	24.0 (CDCl_3_) [20]	231
**1g**	21.8	21.5 (CDCl_3_) [20]	245.1
**1h**	20.4	19.9 (CDCl_3_) [20]	290.1
**1i**	21.2	21.9 (CDCl_3_) [20]	354.1
**1j**	21.7	21.7 (CDCl_3_) [23]	262.1
**4k**	26.7	26.4 (CDCl_3_) [24]	229.2
**4l**	29.8	30.4 (CDCl_3_) [25]	243.1
**4m**	30.6	30.8 (CDCl_3_) [24]	243.1
**2a**	20.8	19.9 (D_2_O) [26]	203.04675 (203.04677 ^c^)
**2b** ^b^	18.8		248.03164 (248.03185 ^c^)
**2c** ^b^	20.3		237.00752 (237.00780 ^c^)
**2d** ^b^	20.4		221.03692 (221.03735 ^c^)
**2e** ^b^	19.6		271.03384 (271.03416 ^c^)
**2f** ^b^	20.8		217.06177 (217.06242 ^c^)
**2g** [27] ^a^	19.6		217.06193 (217.06242 ^c^)
**2h** ^b^	18.1		262.04707 (262.04750 ^c^)
**2i** ^b^	19.0		251.02306 (251.02345 ^c^)
**2j** ^b^	19.5		235.05267 (235.05300 ^c^)
**5k**	23.7	25.5 (CD_3_OD) [28]	201.1
**5l**	27.0	32.3 (CDCl_3_) [29]	215.1
**5m** [30] ^a^	27.5	–	215.1
**3A**	18.9	19.4(D_2_O) [18]	189.1
**3B**	16.6	16.4 (D_2_O) [18]	234.0
**3C**	17.9	18.6 (D_2_O) [18]	223.0
**3D**	18.2	18.5 [31]	207.0
**3E** ^b^	17.4		257.0
**3F**	18.6	18.8 (D_2_O) [18]	203.1
**6K**	21.8	22.1 (DMSO) [32]	173.0
**6L**	25.7	27.8 (CD_3_OD) [33]	187.0
**6M**	26.0	26.7 (DMSO) [34]	187.0

^a 13^C and ^1^H NMR characterization is available in the literature; ^b^ Spectral data are given below; ^c^ Calculated value.

## Supplementary Materials

The following are available online: Figure S1: Concentration profile for the components during the hydrolysis of dimethyl α-hydroxy-4-nitrobenzylphosphonate (**1b**) under optimum conditions. The R^2^ measure of goodness of fit is 0.989. Figure S2: Concentration profile for the components during the hydrolysis of dimethyl α-hydroxy-4-chlorobenzylphosphonate (**1c**) under optimum conditions. The R^2^ measure of goodness of fit is 0.987. Figure S3: Concentration profile for the components during the hydrolysis of dimethyl α-hydroxy-4-fluorobenzylphosphonate (**1d**) under optimum conditions. The R^2^ measure of goodness of fit is 0.965. Figure S4: Concentration profile for the components during the hydrolysis of dimethyl α-hydroxy-4-trifluoromethylbenzylphosphonate (**1e**) under optimum conditions. The R^2^ measure of goodness of fit is 0.988. Figure S5: Concentration profile for the components during the hydrolysis of dimethyl α-hydroxy-4-methylbenzylphosphonate (**1f**) under optimum conditions. The R^2^ measure of goodness of fit is 0.962. Figure S6: Concentration profile for the components during the hydrolysis of diethyl α-hydroxy-4-nitrobenzylphosphonate (**1h**) under optimum conditions. The R^2^ measure of goodness of fit is 0.992. Figure S7: Concentration profile for the components during the hydrolysis of diethyl α-hydroxy-4-chlorobenzylphosphonate (**1i**) under optimum conditions. The R^2^ measure of goodness of fit is 0.992. Figure S8: Concentration profile for the components during the hydrolysis of diethyl α-hydroxy-4-fluorobenzylphosphonate (**1j**) under optimum conditions. The R^2^ measure of goodness of fit is 0.970. Figure S9: Concentration profile for the components during the hydrolysis of diethyl α-phenylethylphosphonate (**4l**) under optimum conditions. The R^2^ measure of goodness of fit is 0.940. Figure S10: Concentration profile for the components during the hydrolysis of diethyl β-phenylethylphosphonate (**4m**) under optimum conditions. The R^2^ measure of goodness of fit is 0.949.
