# Supplementary material for: Optimization and a Kinetic Study on the Acidic Hydrolysis of Dialkyl α-Hydroxybenzylphosphonates
Source: Molecules. 2020 Aug 20;25(17):3793. doi: 10.3390/molecules25173793 (PMC7504060; doi:10.3390/molecules25173793)
Supplement: Supplementary file 1 [file molecules-25-03793-s001.pdf]

molecules

Supplementary Materials

Optimization and a Kinetic Study on the Acidic Hydrolysis of Dialkyl α-Hydroxybenzylphosphonates

Nikoletta Harsági [1], Zita Rádai [1], Áron Szigetvári [2], János Kóti [2] and György Keglevich [1,*]

[1] Department of Organic Chemistry and Technology, Budapest University of Technology and Economics, 1521 Budapest, Hungary
[2] Gedeon Richter Plc., 1475 Budapest, Hungary

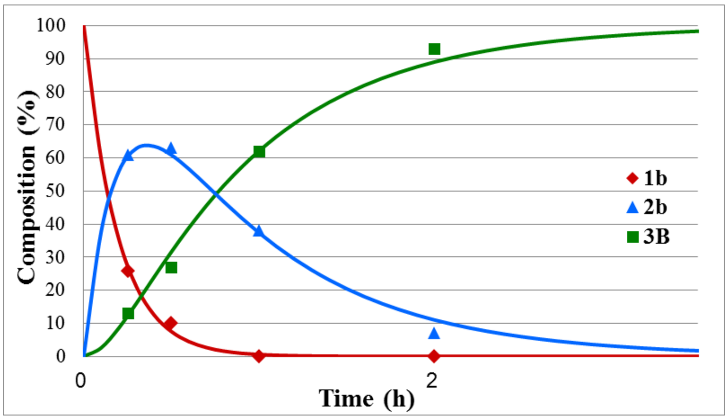

Figure S1. Concentration profile for the components during the hydrolysis of dimethyl α-hydroxy-4-nitrobenzylphosphonate (**1b**) under optimum conditions. The R^2 measure of goodness of fit is 0.989.

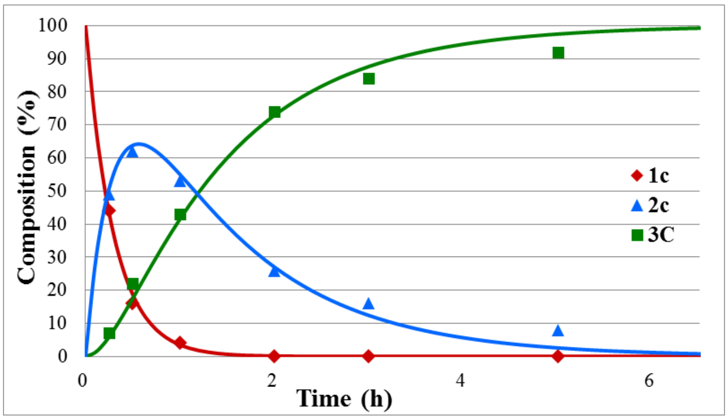

Figure S2. Concentration profile for the components during the hydrolysis of dimethyl α-hydroxy-4-chlorobenzylphosphonate (**1c**) under optimum conditions. The R^2 measure of goodness of fit is 0.987.

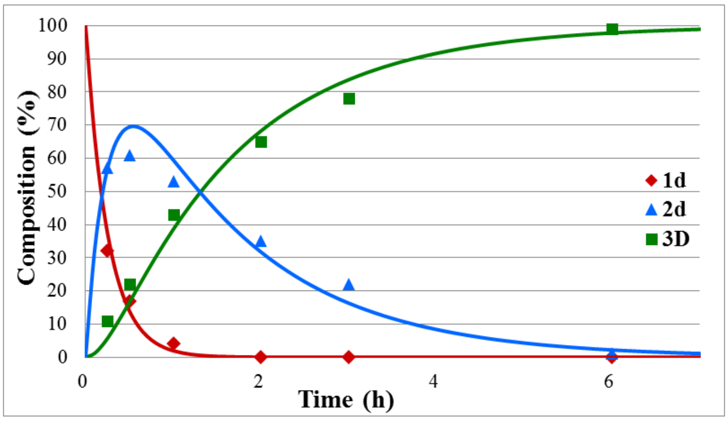

Figure S3. Concentration profile for the components during the hydrolysis of dimethyl α-hydroxy-4-fluorobenzylphosphonate (**1d**) under optimum conditions. The R^2 measure of goodness of fit is 0.965.

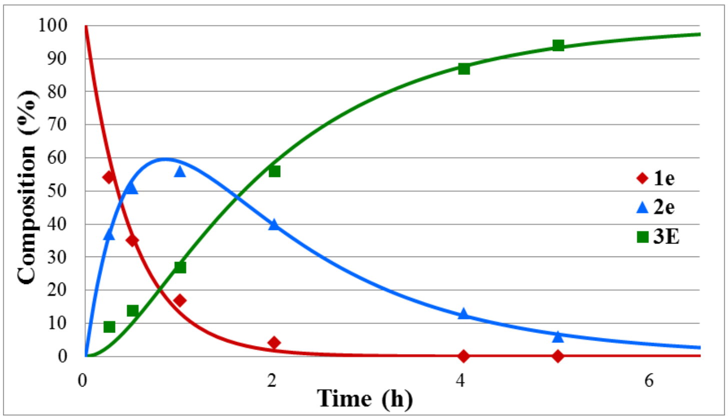

Figure S4. Concentration profile for the components during the hydrolysis of dimethyl α-hydroxy-4-trifluoromethylbenzylphosphonate (**1e**) under optimum conditions. The R^2 measure of goodness of fit is 0.988.

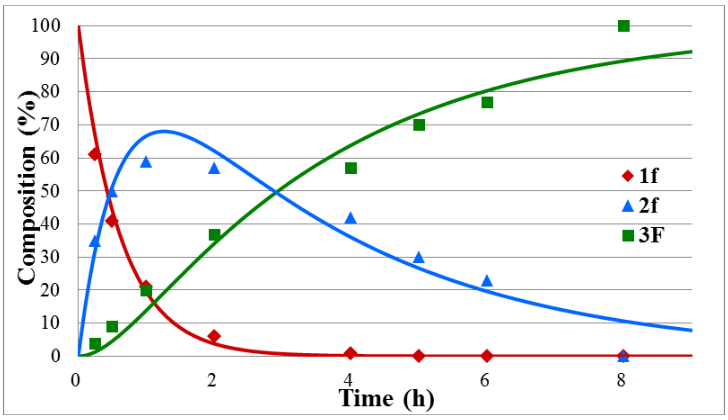

Figure S5. Concentration profile for the components during the hydrolysis of dimethyl α-hydroxy-4-methylbenzylphosphonate (**1f**) under optimum conditions. The R^2 measure of goodness of fit is 0.962.

molecules

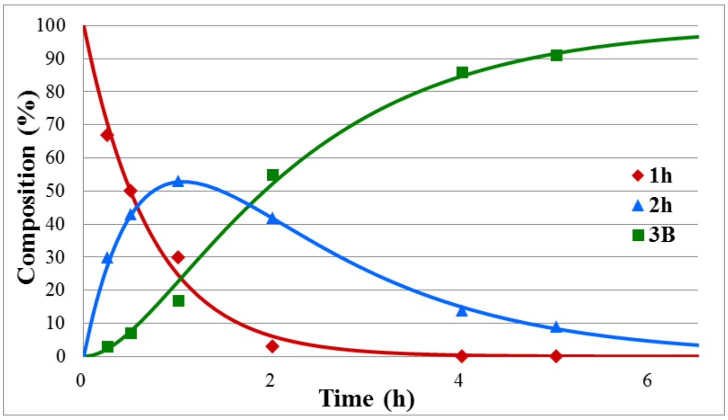

Figure S6. Concentration profile for the components during the hydrolysis of diethyl α-hydroxy-4-nitrobenzylphosphonate (**1h**) under optimum conditions. The R^2 measure of goodness of fit is 0.992.

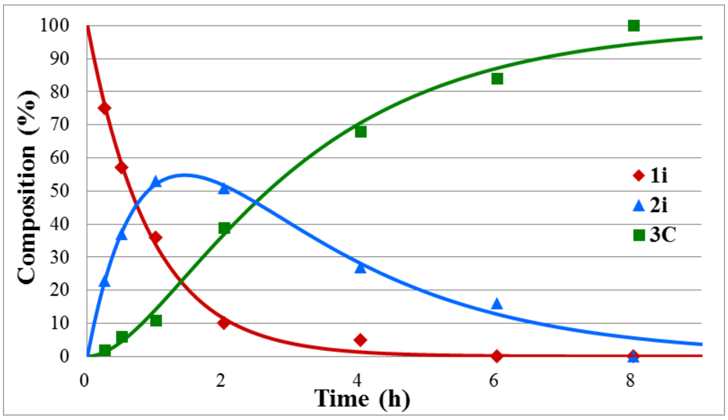

Figure S7. Concentration profile for the components during the hydrolysis of diethyl α-hydroxy-4-chlorobenzylphosphonate (**1i**) under optimum conditions. The R^2 measure of goodness of fit is 0.992.

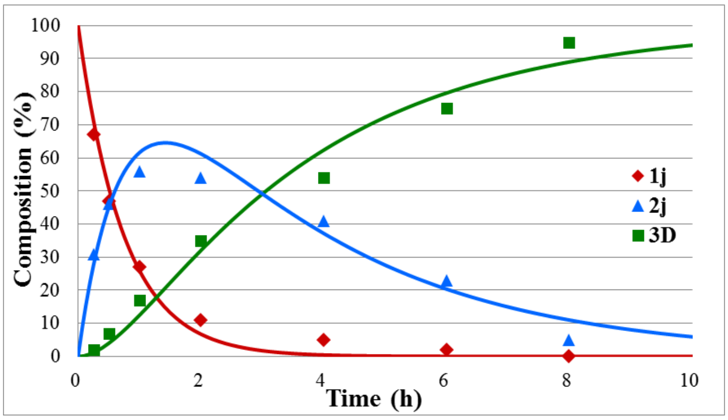

Figure S8. Concentration profile for the components during the hydrolysis of diethyl α-hydroxy-4-fluorobenzylphosphonate (**1j**) under optimum conditions. The R^2 measure of goodness of fit is 0.970.

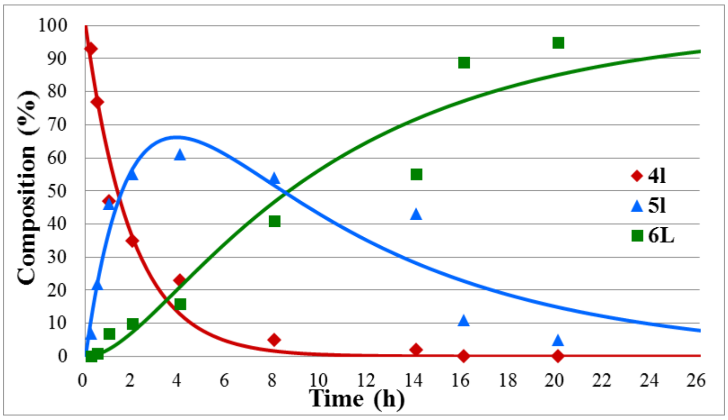

Figure S9. Concentration profile for the components during the hydrolysis of diethyl α-phenylethylphosphonate (**4l**) under optimum conditions. The R^2 measure of goodness of fit is 0.940.

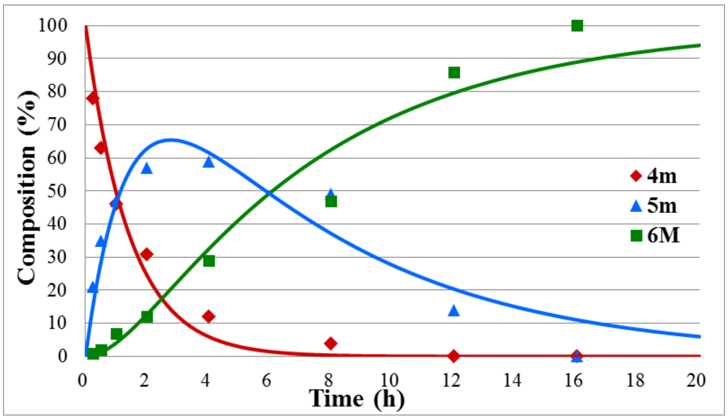

Figure S10. Concentration profile for the components during the hydrolysis of diethyl β-phenylethylphosphonate (**4m**) under optimum conditions. The R^2 measure of goodness of fit is 0.949.
